# Recent Advances in Nonfullerene Acceptor‐Based Layer‐by‐Layer Organic Solar Cells Using a Solution Process

**DOI:** 10.1002/advs.202201876

**Published:** 2022-07-06

**Authors:** Min Hun Jee, Hwa Sook Ryu, Dongmin Lee, Wonho Lee, Han Young Woo

**Affiliations:** ^1^ Department of Chemistry KU‐KIST Graduate School of Converging Science and Technology Korea University Seoul 02841 Republic of Korea; ^2^ Department of Polymer Science and Engineering Department of Energy Engineering Convergence Kumoh National Institute of Technology Gumi Gyeongbuk 39177 Republic of Korea

**Keywords:** layer‐by‐layer, nonfullerene acceptors, organic photovoltaics, pseudo‐planar heterojunction

## Abstract

Recently, sequential layer‐by‐layer (LbL) organic solar cells (OSCs) have attracted significant attention owing to their favorable p–i–n vertical phase separation, efficient charge transport/extraction, and potential for lab‐to‐fab large‐scale production, achieving high power conversion efficiencies (PCEs) of over 18%. This review first summarizes recent studies on various approaches to obtain ideal vertical D/A phase separation in nonfullerene acceptor (NFAs)‐based LbL OSCs by proper solvent selection, processing additives, protecting solvent treatment, ternary blends, etc. Additionally, the longer exciton diffusion length of NFAs compared with fullerene derivatives, which provides a new scope for further improvement in the performance of LbL OSCs, is been discussed. Large‐area device/module production by LbL techniques and device stability issues, including thermal and mechanical stability, are also reviewed. Finally, the current challenges and prospects for further progress toward their eventual commercialization are discussed.

## Graphical abstract

Layer‐by‐layer (LbL) organic solar cells (OSCs) have attracted growing attention, showing photovoltaic performance comparable to their bulk heterojunction counterparts. Recent developments on nonfullerene acceptor‐based LbL OSCs are discussed by considering vertical p–i–n morphology control, exciton diffusion/separation, and large area device fabrication.

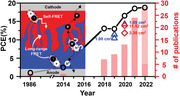

## 1 Introduction

During last several decades, organic solar cells (OSCs) have been extensively studied as potential clean and renewable energy harvesters along with their advantages of facile tuning of optical and electrical properties of photoactive materials, mechanical flexibility, light weight, and solution processability at room temperature.^[^ ^1^, ^2^, ^3^, ^4^ ^]^ Tang et al. reported the first donor (D)/acceptor (A) planar heterojunction (PHJ) OSCs by vacuum deposition with a power conversion efficiency (PCE) of ≈1% in 1986, but such a device structure limited the photovoltaic performance due to short exciton diffusion length (*L*_D_) of photoactive materials and the limited D/A interface.^[^ ^5^ ^]^ In 1995, the concept of bulk heterojunction (BHJ) morphology was introduced by Heeger et al., which enables efficient exciton dissociation by increasing the D/A interfaces.^[^ ^6^ ^]^ With this genius approach, BHJ devices have been a main research focus in the field of OSCs and the PCEs of over 18–19% have been successfully achieved recently.^[^ ^7^, ^8^, ^9^, ^10^ ^]^ However, the control of ideal BHJ morphology is not practically possible and is even more difficult in the large‐scale fabrication of OSCs. The intermixed state in the BHJ structure is beneficial for effective charge generation by creating a topologically disordered film but is not conducive to efficient charge carrier transport. To maximize the charge collection efficiency at the electrodes, the desired vertical phase separation with enrichment of the donor and acceptor phases near the anode and cathode, respectively, is needed, but difficult to achieve in BHJ OSCs. Furthermore, BHJ morphology is often produced as a kinetically trapped state by rapid evaporation of the solvent, which is not thermodynamically stable and gradually changes into a more stable state with larger phase separation during device operation. These issues make the large‐scale industrialization of BHJ OSCs difficult.

**Figure 1:**
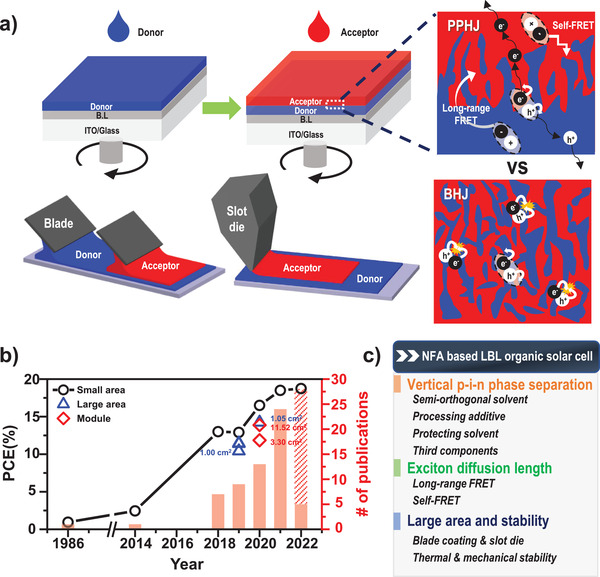
a) Sequential LbL processes (spin casting, blade coating, and slot die coating) and evolution of p–i–n morphology. b) Recent developments in small‐ and large‐area solution‐processed LbL OSCs. c) Main discussion topics of this review.

As an alternative approach, sequential layer‐by‐layer (LbL)‐processed pseudo‐planar heterojunction (PPHJ) OSCs have attracted significant attention for solving the above concerns. Although vacuum deposition^[^ ^5^, ^11^ ^]^ and stamping techniques^[^ ^12^, ^13^ ^]^ have been reported, sequential solution processing is currently the mainstream method used for producing LbL PPHJ devices. Unlike solution‐processed BHJ devices, LbL strategies can form a pseudo‐bilayer (p–i–n) structure in the active layer, where proper vertical phase separation can be controlled to optimize charge transport and extraction at the corresponding electrodes. In addition, the film thickness and crystalline morphology of D and A layers in PPHJ can be controlled independently by using solvent additives and thermal annealing. Consequently, the PPHJ geometry enables the facile control of exciton generation and dissociation, and can reduce geminate and nongeminate charge carrier recombination losses (**Figure** **1**a).

In LbL‐processed p–i–n OSCs with sequential deposition of D and A layers using orthogonal (or semiorthogonal) solvents, the D/A interfaces can be limited compared with BHJ films, making them inefficient for exciton dissociation. Several previous studies have revealed that nonfullerene acceptors (NFAs) have longer *L*_D_s than fullerenes, enabling optimal domain sizes of 20–50 nm in BHJ blends.^[^ ^14^, ^15^, ^16^ ^]^ Consequently, the enhanced *L*_D_ of NFAs may also obviate the need for BHJ morphology and is beneficial for further optimizing PHJ and/or PPHJ devices. In 2018, Friend et al. and Hou et al. successfully demonstrated a significant breakthrough in the development of high‐performing LbL OSCs based on NFAs.^[^ ^17^, ^18^ ^]^ After these pioneering works, many research groups investigated sequential LbL‐processed PPHJ OSCs extensively, including polymer donors and small molecular acceptor pairs, all‐polymer D/A pairs, ternary and quaternary mixtures, processing additives, and posttreatments to further optimize vertical D/A phase separation and device performance. Benefiting from these efforts, significant progress has been made recently in LbL OSCs to show PCEs of over 18% (Figure 1b and **Table** **1**).^[^ ^19^, ^20^, ^21^ ^]^ In addition, the charge dynamics of LbL OSCs have been studied in comparison to those of BHJ OSCs, including exciton separation and charge recombination via the transient absorption spectroscopy technique. The lower energy loss has also been claimed to be an advantage of LbL PPHJ devices. Furthermore, the device stability, including thermal and mechanical stability, has been widely studied. Owing to these advantages, sequential LbL processing demonstrates great potential for the large‐area roll‐to‐roll (R2R) mass production of PPHJ OSCs. For example, Min et al. successfully fabricated a large‐area solar module of 11.52 cm^2^ with a PCE of over 11% by blade coating.^[^ ^22^ ^]^ Significant efforts have been dedicated, and noticeable advances have been made in LbL PPHJ OSCs based on NFAs over the last several years. Therefore, a review of recent advances in the field of sequential LbL‐processed OSCs, highlighting important scientific breakthroughs, would be of great significance.

**Table 1:** Photovoltaic properties of NFAs‐based LbL OSCs

Active layer	Processing condition	*V*_OC_ [V]	*J*_SC_ [mA cm^‐2^]	FF [%]	PCE [%]	Refs.
Binary system						
*α*‐6T(**1‐1**)/SubNc(**2‐1**, A_1_)/SubPc(**2‐2**, A_2_)	ITO/PEDOT:PSS/D (VD, 60 nm)/A_1_ (VD, 12 nm)/A_2_ (VD, 18 nm)/BCP/Ag	0.96	14.55	61.00	8.40	^[^ ^29^ ^]^
PIDSe‐DFBT(**2‐3**)/P3HT(**1‐2**)	ITO/ZnO/A (*o*‐DCB)/D (DCM:CB(100:10))/MoO_3_/Ag	1.15	4.09	0.52	2.45	^[^ ^30^ ^]^
PBDB‐TFS1(**1‐3**)/IT‐4F(**2‐4**)	ITO/PEDOT:PSS/D (CB,50 nm)/A (THF:*o*‐DCB(100:5))/PFN‐Br/Al	0.90	20.30	71.00	13.00	^[^ ^18^ ^]^
PNNT(**2‐5**)/PBDTT‐FTTE(**1‐4**)	ITO/PFN/A (*o*‐DCB)/D (*o*‐DCB)/MoO_3_/Ag	0.74	10.70	52.00	4.10	^[^ ^31^ ^]^
P(NDI2OD‐T2)(**2‐6**)/PTB7(**1‐5**)	ITO/ZnO/A (*o*‐DCB:CB(2:1))/D (DCM:CB(2:1))/MoO_3_/Ag	0.74	7.61	54.10	3.05	^[^ ^32^ ^]^
PBDB‐T(**1‐6**)/NCBDT(**2‐7**)	ITO/PEDOT:PSS/D (CF, 45 nm)/A (DCM, 50 nm)/PDINO/Al	0.82	19.45	62.90	10.04	^[^ ^17^, ^33^ ^]^
PTFB‐O(**1‐7**)/ITIC‐Th(**2‐8**)	ITO/PEDOT:PSS/D (CB, 50 nm)/A (THF, 50 nm)/PFN‐Br/Ag	0.91	17.50	74.00	11.80	^[^ ^34^ ^]^
PBDB‐T(**1‐6**)/ITIC(**2‐9**)	ITO/ZnO/D (CB)/A (*o*‐XY)/MoO_3_/Ag	0.86	15.30	50.50	6.70	^[^ ^35^ ^]^
PBDB‐T(**1‐6**)/FPDI‐BT1(**2‐10**)	ITO/PEDOT:PSS/D (*o*‐DCB)/A (o‐DCB:CB(1:1))/ZnO/Al	0.96	11.63	61.80	6.93	^[^ ^36^ ^]^
N2200(**2‐6**)/PBDB‐T(**1‐6**)	ITO/ZnO/A (CB, 60 nm)/D (CF, 50 nm)/MoO_3_/Ag	0.90	15.33	68.70	9.52	^[^ ^37^, ^38^ ^]^
FTAZ(**1‐8**)/IT‐M(**2‐11**)	ITO/PEDOT:PSS/D (LM)/A (2‐MeTHF)/PFN‐Br/Al	0.96	18.30	70.00	12.20	^[^ ^39^ ^]^
PM6(**1‐9**)/Y6‐BO(**2‐12**)	ITO/PEDOT:PSS/D (CF)/A (CF with 0.25 vol% CN)/PNDIT‐F3N/Ag	0.85	26.20	77.50	17.20	^[^ ^40^ ^]^
PBDB‐T‐2F(**1‐9**)/IT‐4F(**2‐4**)	ITO/PEDOT:PSS/D (CB:ODT (99:1))/A (DCM)/ZnO/Ag	0.90	16.20	69.00	10.10	^[^ ^41^ ^]^
PTB7‐Th(**1‐10**)/IEICO‐4F(**2‐13**)	ITO/PEDOT:PSS/D (*o*‐XY, 60 nm)/A (*o*‐XY:n‐butanol (75:25), 70 nm)/PNDIT‐F3N‐Br/Ag	0.66	20.00	62.60	8.30	^[^ ^42^ ^]^
PBDB‐T(**1**‐**6**)/PYT(**2‐14**)	^a)^ITO/ZnO/D (CB, 50 nm)/A (CB, 40 nm)/MoO_3_/Ag	0.72	23.45	66.40	11.26	^[^ ^43^ ^]^
PT2(**1‐11**)/Y6(**2‐15**)	ITO/PEDOT:PSS/D (CB)/A (CF with 1 vol% DIO)/PNDIT‐F3N‐Br/Ag	0.83	26.70	74.40	16.50	^[^ ^44^ ^]^
D18(**1‐12**)/(BTIC‐)BO‐4Cl(**2‐16**)	ITO/PEDOT:PSS/D (CF, 40 nm)/A (Tol with 0.5 vol% DIO, 50 nm)/PNDIT‐F3N/Ag	0.86	26.32	77.66	17.60	^[^ ^15^ ^]^
PM6(**1‐9**)/IT‐4F(**2‐4**)	ITO/PEDOT:PSS/D (CB)/A (CB with 1 vol% DIO)/PDINO/Al	0.86	20.98	75.90	13.70	^[^ ^45^ ^]^
J60(**1‐13**)/TBDPDI‐C_5_(**2‐17**)	ITO/ZnO/D (CB)/A (CB)/MoO_3_/Ag	0.97	10.57	59.62	5.91	^[^ ^46^ ^]^
PM7(**1‐14**)/IT‐4F(**2‐4**)	ITO/PEDOT:PSS/D (CB)/A (CF)/PFN‐Br/Al	0.88	20.06	70.16	12.38	^[^ ^47^ ^]^
IT‐4F(**2‐4**)/PM6(**1‐9**)	^b)^ITO/ZnO/A (DCM)/D (CB with 1 vol% DIO)/MoO_3_/Ag	0.86	14.30	61.00	7.47	^[^ ^48^ ^]^
PNTB6‐Cl(**1‐15**)/N3(**2‐18**)	ITO/PEDOT:PSS/D (CB, 70 nm)/A (CF with 0.5 vol % DIO, 45 nm)/PNDIT‐F3N/Ag	0.86	26.58	77.30	17.59	^[^ ^49^ ^]^
PBDB‐T‐2F(**1‐9**)/ITIC‐Th1(**2‐19**)	ITO/PEDOT:PSS/D (CB:ODT (100:0.5), 50 nm)/A (DCM, 35 nm)/ZnO/Al	0.94	16.90	69.00	11.00	^[^ ^16^ ^]^
PBTZT‐stat‐BDTT‐8(**1‐16**)/Cl‐Cl_6_BsubPc(**2‐20**)	ITO/PEDOT:PSS/D (*o‐*DCB, 30 nm)/A (VD, 20 nm) /BCP/Ag	0.82	2.98	66.00	1.60	^[^ ^50^ ^]^
PBDB‐T‐2F(**1‐9**)/Y6(**2‐15**)	ITO/PEDOT:PSS/D (CF, 60 nm) /A (CF, 40 nm)/ZnO/Au/Ag	0.83	22.30	68.80	12.24	^[^ ^51^ ^]^
PM6(**1‐9**)/Y6(**2‐15**)	ITO/PEDOT:PSS/D (CF with 0.5 wt% DDO)/A (CF with 0.5 vol % CN) /PDINO/Al	0.85	25.51	77.45	16.93	^[^ ^52^ ^]^
PM6(**1‐9**)/Y6(**2‐15**)	ITO/PEDOT:PSS/CS/D (CB)/A (CF)/PDINO/Ag	0.85	26.58	78.60	17.84	^[^ ^53^ ^]^
D18(**1‐12**)/n‐octane/N3(**2‐18**)	ITO/PEDOT:PSS/D (CF)/A (CF) & 120 nm/PDINO/Al	0.83	27.79	75.61	17.52	^[^ ^54^ ^]^
PBDB‐T(**1‐6**)/PYT(**2‐14**)	^a)^ITO/PEDOT:PSS/D (CF)/ A (CF with 2.5 vol% CN)/PFN‐Br/Ag	0.89	22.95	73.88	15.10	^[^ ^55^ ^]^
PM6(**1‐9**)/TB‐4F(**2‐21**)	ITO/PEDOT:PSS/D (CF)/A (CF with 0.25 vol% CN)/PDINO/Al (Under 1000 lux LED)	0.69	0.12	77.89	21.05	^[^ ^56^ ^]^
PM6(**1‐9**)/Y6(**2‐15**)	^c)^ITO/ZnO:PEI/D (CB)/A (CF)/ MoO_3_/Ag TA at 135 ℃ for 10 min	0.80	25.28	71.30	14.42	^[^ ^57^ ^]^
DTDCTB(**1‐17**)/CBD(**2‐22**)	ITO/MoO_3_/D (VD, 10 nm)/A (VD, 20 nm)/BPhen/Al	0.78	2.93	37.58	0.86	^[^ ^58^ ^]^
PTzNDI‐T(**2‐23**)/J52‐Cl(**1‐18**)	ITO/ZnO/A (TMB)/D (CB)/MoO_3_/Ag	0.94	10.10	64.06	6.08	^[^ ^59^ ^]^
P2F‐EHp(**1‐19**)/M4‐4F(**2‐24**)	ITO/PEDOT:PSS/D (CF:DBE (100:2), 50 nm)/A (DCM, 50 nm)/PFNDI‐Br/Ag	0.83	25.56	67.14	14.21	^[^ ^60^ ^]^
PM6(**1‐9**)/Y6(**2‐15**)	^a)^ITO/PEDOT:PSS/D (CF)/A (CF)/PDINN/Ag	0.84	26.22	73.52	16.17	^[^ ^61^ ^]^
PM6(**1‐9**)/BO‐4F(**2‐25**)	ITO/PEDOT:PSS/D (*o*‐XY)/A (THF)/PDINN/Ag	0.82	26.20	74.30	16.00	^[^ ^62^ ^]^
PM6(**1‐9**)/L8‐BO(**2‐26**)	ITO/PEDOT:PSS/D (CB with 0.5 wt% wax)/A (CF with 0.3 vol% DIM) /PNDIT‐F3N/Ag	0.89	26.11	80.60	18.74	^[^ ^21^ ^]^
PBDB‐T(**1‐6**)/PYT(**2‐14**)	ITO/PEDOT:PSS/D (CF)/A (CF with 3.0 vol% CN)/PFN‐Br/Ag	0.91	23.07	77.00	16.05	^[^ ^63^ ^]^
Ternary and quaternary systems						
PBDB‐T(**1‐6**):ITIC(**2‐9**)(1:1)/IDIC(**2‐27**)	ITO/ZnO/D (DCB:CF (100:10) with 3 vol% DIO)/A (CF)/MoO_3_/Ag	0.90	16.00	72.30	10.40	^[^ ^64^ ^]^
PDCBT(**1‐20**)/PC_71_BM(**2‐28**):ITIC(**2‐9**)(1:1)	ITO/PEDOT:PSS/D (CB)/A (DCM:DIM(99:1))/LiF/Al	0.87	10.12	71.00	6.22	^[^ ^65^ ^]^
PM6(**1‐9**)/Y6(**2‐15**):PC_71_BM(**2‐28**)(1:0.2)	ITO/PEDOT:PSS/D (CB)/A (CF with 0.5 vol% DIO)/PDINO/Ag	0.83	26.60	77.10	17.00	^[^ ^66^ ^]^
PTB7‐Th(**1‐10**, D_1_):IEICO‐4F(**2‐13**, A_1_)/PBDB‐T‐2F(**1‐9**, D_2_):IT‐4F(**2‐4**, A_2_)	ITO/ZnO/D_1_: A_1_ (CB)/D_2_:A_2_ (CB)/ MoO_3_/Ag	0.77	23.94	69.81	12.86	^[^ ^67^ ^]^
PffBT4T‐2OD(**1‐21**)/Y6(**2‐15**):FBR(**2‐29**)(0.8:0.2)	ITO/PEDOT:PSS/D (*o*‐XY, 60 nm)/A (*o*‐XY, 50 nm)/PFN‐Br/Ag	0.83	26.30	75.60	16.40	^[^ ^68^ ^]^
PM6(**1‐9**)/IT‐4F(**2‐4**):F8‐IC(**2‐30**)(0.55:0.45)	ITO/PEDOT:PSS/D (CB)/A (CB) /PDINO/Al	0.79	25.60	69.80	14.20	^[^ ^69^ ^]^
PBDB‐T(**1‐6**, D_1_):IT‐M(**2‐11**, A_1_)/PBDB‐T(**1‐6**, D_2_):FOIC(**2‐31**, A_2_)	^a)^ITO/ZnO/D_1_:A_1_ (CB, 15 nm)/ D_2_:A_2_ (CB, 100 nm)/MoO_3_/Ag	0.75	24.66	63.57	11.86	^[^ ^70^ ^]^
PM6(**1‐9**):Y6‐Se‐4Cl(**2‐32**) (1:1.2)/PM6(**1‐9**):Y6(**2‐15**)(1:1)	ITO/PEDOT:PSS/D (CF with 0.5 vol% CN, 100 nm)/A (CF with 0.5 vol% CN, 20 nm)/MoO_3_/Ag	0.84	26.30	73.00	16.10	^[^ ^71^ ^]^
PTO3(**1‐22**, D_1_)/PBDB‐TF(**1‐9**, D_2_):BTP‐eC9(**2‐33**, A_2_)/NDI‐i8(**2‐34**, A_1_)	ITO/PEDOT:PSS/D_1_ (*o*‐DCB)/D_2_:A_2_ (CF)/A_1_(VD)/PF3N/Ag	0.87	26.60	80.30	18.50	^[^ ^19^ ^]^
PM6(**1‐9**)/N3(**2‐18**):PC_71_BM(**2‐28**)(0.96:0.24)	ITO/PEDOT:PSS/D (CF, 50 nm)/A (CF with 0.5 vol% CN, 49 nm)/PNDIT‐F3N/Ag	0.84	26.49	78.20	17.42	^[^ ^72^ ^]^
PTB7‐Th(**1‐10**)/IEICO‐4F(**2‐13**):PC_71_BM(**2‐28**)(0.8:0.2)	ITO/PEDOT:PSS/D (CF)/A (DCM with 4 vol% CN)/TiO_2_/Al	0.72	25.40	62.00	11.00	^[^ ^73^ ^]^
PM6(**1‐9**)/BO‐4Cl(**2‐16**):BTP‐S2(**2‐35**)(0.75:0.25)	ITO/PEDOT:PSS/D (CF with 0.25 vol% DIO)/A (CF)/PFN‐Br/Ag	0.86	27.14	78.04	18.16	^[^ ^20^ ^]^
PBDB‐T‐2F(**1‐9**)/PBDB‐T‐2F(**1‐9**):Y6(**2‐15**)(0.1:0.9)/	ITO/PEDOT:PSS/D (CB)/A (CF)/PFN‐Br/Al	0.83	26.52	70.88	15.41	^[^ ^74^ ^]^
PM6(**1‐9**)/Y6(**2‐15**):TF1(**2‐36**)(1.1:0.1)	ITO/PEDOT:PSS/D (CB)/A (CF with 0.5 vol% CN)/PDINO/Al	0.87	25.89	75.08	16.85	^[^ ^75^ ^]^
PM6(**1‐9**)/F8‐IC(**2‐30**):IT‐4F(**2‐4**)(0.6:0.4)	ITO/PEDOT:PSS/D (CB) /A (CB with 0.5 vol% DIO)/PDINO/Al	0.77	25.20	70.60	13.80	^[^ ^76^ ^]^

All device fabrications are based on spin‐coating except for ^a)^blade coating;^b)^film transfer; ^c)^slot die coating.

Abbreviations: *VD* vacuum deposition, *DBE* dibenzyl ether, *TMB* 1,2,3‐trimethylbenzene, *LM* (R)‐(+)‐limonene, *2‐MeTHF* 2‐methyltetrahydrofuran, *Tol* toluene, *DIM* diiodomethane, *ODT* 1,8‐octanedithol, *BCP* bathocuproine, *PFN‐Br* poly(9,9‐bis(3′‐(*N,N*‐dimethyl)‐*N*‐ethylammoinium‐propyl‐2,7‐fluorene)‐alt‐2,7‐(9,9‐dioctylfluorene))dibromide, *PNDIT‐F3N* poly[[2,7‐bis(2‐ethylhexyl)‐1,2,3,6,7,8‐hexahydro‐1,3,6,8‐tetraoxobenzo[lmn][3,8‐phenanthroline‐4,9‐diyl]‐2,5‐thiophenediyl[9,9‐bis[3‐(dimethylamino)propyl]‐9H‐fluorene‐2,7‐diyl]‐2,5‐thiophenediyl], *CS* cyanostar, *BPhen* 4,7‐diphenyl‐1,10‐phenanthroline, *PFNDI‐Br* poly[(9,9‐bis(3′‐((*N,N*‐dimethyl)‐N‐ethylammonium)propyl)‐2,7‐fluorene)‐alt‐5,5′‐bis(2,2′‐thiophene)‐2,6‐naphthalene‐1,4,5,8‐tetracaboxylic‐*N,N′*‐di(2‐ethylhexyl)imide]dibromide, *PDINN N,N′*‐bis{3‐[3‐(dimethylamino)propylamino]propyl}perylene‐3,4,9,10‐tetracarboxylic diimide, *PF3N* poly[(9,9‐bis(3′‐(*N,N*‐dimethylamino)propyl)‐2,7‐fluorene)‐alt‐5,5′‐bis(2,2′‐thiophene)‐2,6‐naphthalene‐1,4,5,8‐tetracaboxylic‐*N,N′*‐di(2‐ ethylhexyl)imide].

This review summarizes recent studies on NFA‐based LBL OSCs by solution processing. To access other results, including fullerene‐based LbL OSCs, readers can refer to previous reviews where various materials including fullerene derivatives and their sequential LbL processing methods (i.e., vacuum deposition, hybrid spin casting/evaporation, stamping/lamination, and nanoimprinting) have been well‐documented.^[^ ^23^, ^24^, ^25^, ^26^, ^27^, ^28^ ^]^ First, recent developments in PPHJ morphological control are summarized with a particular emphasis on vertical p–i–n (or n–i–p) phase separation by proper solvent selection, processing additives, protecting solvent treatment, thermal treatment, temperature‐dependent aggregation structures, and ternary components. Second, the longer *L*_D_ of NFAs (compared to fullerene derivatives) is discussed in terms of self‐Förster resonance energy transfer (FRET) between acceptor molecules in the A phase to increase flexibility in the morphological control for high‐performance LbL OSCs. Third, large‐area devices and LbL OSC modules are discussed, along with their significant advantages of voltage loss and device stability, including thermal and mechanical stability (Figure 1c). Finally, the current challenges and prospects for further optimization of LbL‐processed PPHJ OSCs and their successful industrialization are discussed.

## 2 Vertical p–i–n Morphology Control

### 2.1 BHJ versus LbL: Advantages of LbL Approach in Optimizing Processing Conditions

BHJ structures, which are generally fabricated by blending donor and acceptor materials in the same solvent, have been a dominant configuration for OSCs because they can create sufficient D/A interfaces for exciton dissociation. Despite their success, conventional BHJ structures have obvious limitations in terms of controlling their ideal morphology and morphological stability. First, charge recombination at the cathode and anode is unavoidable to some extent, as donor and acceptor phases are usually distributed randomly in a vertical direction within the BHJ structures; consequently, charges are not collected at the cathodes and anodes that have interfaces with the donor (responsible for hole transport) and acceptor (electron transport) phases, respectively. In particular, the *π*‐conjugated chemical structures of NFAs and polymer donors are similar, resulting in a low Flory‐Huggins interaction parameter *χ*, which makes it more difficult to obtain the desired D/A vertical phase distribution. Second, optimizing the BHJ morphology is very sensitive to the material structure and processing conditions, such as the blend ratio, solvent, solvent additives, and thermal annealing. Predicting and controlling the BHJ morphology, which is dependent on the processing conditions, also remains a challenge because the solution process of the mixture of donor and acceptor materials leads to complicated dynamics and kinetics during the morphological evolution.^[^ ^77^ ^]^ Finally, BHJ morphology is not a thermodynamically stable state, which causes a decrease in PCE due to morphological changes during device operation.

The LbL structures can overcome the aforementioned limitations of BHJ structures. LbL films are fabricated independently on the bottom electrode and are composed of a photoactive layer (donor) followed by another layer (acceptor) in the case of conventional OSCs. Therefore, it is easy to produce the desired D/A vertical phase separation in which the donor and acceptor phases are abundant near the anode and cathode, respectively (Figure 1a). This is beneficial for charge collection at the corresponding electrodes. LbL structures can create an intermixed phase as a BHJ morphology in the middle part of LbL structures, in which the volume and domain size of the intermixed phase are mainly controlled by the choice of solvent and/or solvent additives when depositing a second layer, as discussed in detail in the following section. The thickness and crystallinity of each layer can also be controlled separately in the LbL structure, thereby simplifying the optimization process for device fabrication compared with BHJ structures. Moreover, the LbL structures are possibly close to a thermodynamically stable state, which is conducive to improving the long‐term stability of OSCs.

### 2.2 Importance of Solvent Choice for Desired LbL Morphology

A critical task for producing high‐performance LbL OSCs is selecting a suitable processing solvent when depositing the second layer. The solvent should be orthogonal but slightly dissolve the first layer to form sufficient D/A interfaces while achieving the desired vertical distribution of the two phases. Therefore, most of the reports are based on a conventional device architecture consisting of an NFA layer on top of a polymer donor layer because entangled structures of polymer donors usually exhibit high solvent resistance against various solvents. As second layer solvent dissolving NFAs, low boiling point solvents that are quasi‐orthogonal to the underlying layers, such as dichloromethane (DCM), tetrahydrofuran (THF), and chloroform (CF), are usually considered.^[^ ^15^, ^17^, ^18^, ^33^, ^34^, ^39^, ^60^ ^]^

**Figure 2:**
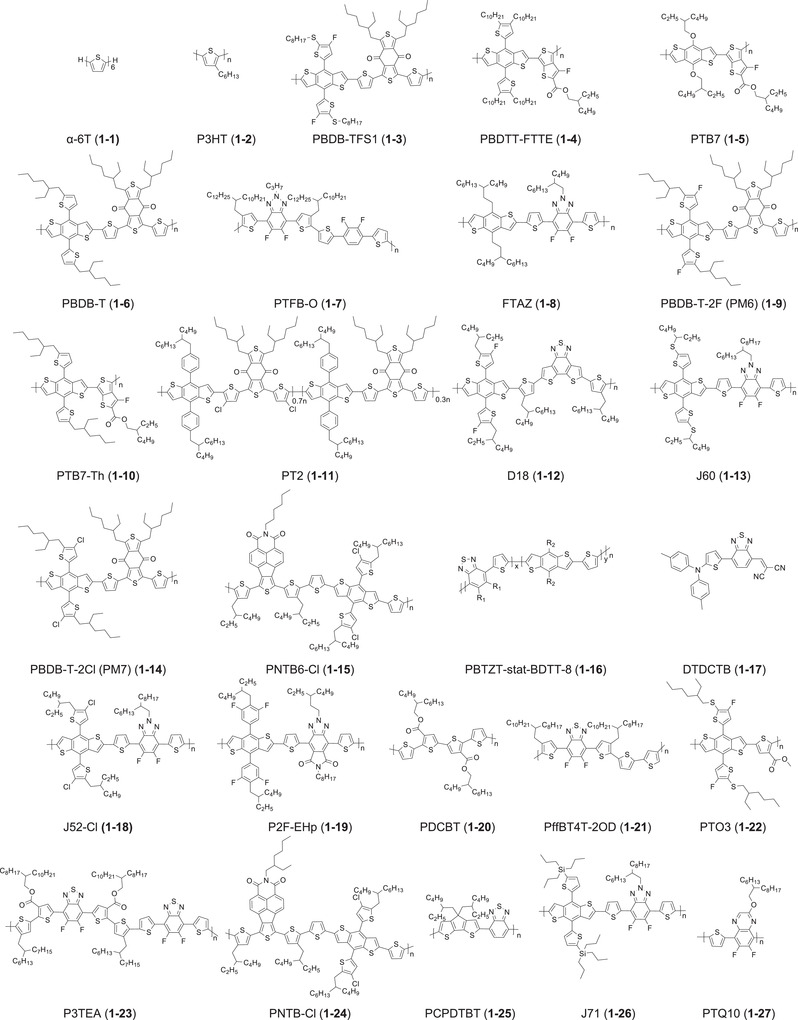
Molecular structures of donor materials discussed in this review.

**Figure 4:**
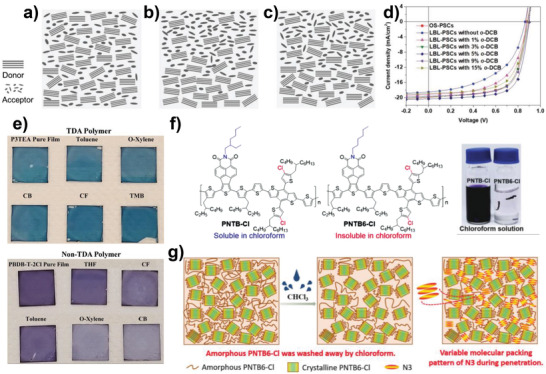
Schematic diagrams of film morphology in a) BHJ OSCs, b) LbL OSCs without *o*‐DCB, and c) LbL OSCs with 5% *o*‐DCB. d) Current density–voltage (*J–V*) curves of LbL OSCs with different contents of *o*‐DCB. Reproduced with permission.^[^ ^18^ ^]^ Copyright 2018, . e) Photographs for TDA (P3TEA) and non‐TDA (PBDB‐T‐2Cl) polymer‐based pristine films after spin‐coating several solvents on top of them. Reproduced with permission.^[^ ^34^ ^]^ Copyright 2019, Wiley‐VCH. f) Different solubility of PNTB‐Cl and PNTB6‐Cl in CF and g) schematic for their morphological evolution by deposition of N3 in CF. Reproduced with permission.^[^ ^49^ ^]^ Copyright 2021, Royal Society of Chemistry.

In 2018, the first successful examples of high‐performing LbL OSCs based on NFAs were reported simultaneously by two groups. Friend et al. fabricated conventional LbL OSCs of indium tin oxide (ITO)/poly(3,4‐ethylenedioxythiophene) (PEDOT):polystyrene sulfonate (PSS)/polymer donor/NFA/2,9‐bis[3‐(dimethyloxidoamino)propyl]anthra(2,1,9‐def:6,5,10‐d'e′f′)diisoquinoline‐1,3,8,10(2H,9H)‐tetrone (PDINO)/Al, in which PBDB‐T (**1‐6**), which is widely used as a benchmark electron donor, and a benzodi(cyclopentadithiophene)‐based NFA, NCBDT (**2‐7**) were selected as donor and acceptor layers, respectively.^[^ ^17^ ^]^ For discussing various donor and acceptor materials, their abbreviations have been directly used because full names are often difficult to define clearly for complex D and A structures. The molecular structures of the donors and acceptors discussed in this review are shown in **Figures** **2** and **3**, respectively. NCBDT was spin‐cast using DCM as the processing solvent following the deposition of the PBDB‐T film with a CF solvent. They optimized the photovoltaic performance by varying only the PBDB‐T and NCBDT layer thicknesses without any additional treatments such as thermal annealing, solvent annealing, and the use of solvent additives or cosolvent; the optimal thickness of the active layer was 90 nm (45 nm for PBDB‐T + 50 nm for NCBDT). They achieved high PCEs of 10.19%, which is comparable to BHJ OSCs showing 10.04% PCEs. DCM dissolves NCBDT well but only partially dissolves PBDB‐T. Therefore, it was assumed that polymer reorganization could occur during the second layer deposition, likely inducing intermixing between PBDB‐T and NCBDT. Around the same time, Hou et al. also demonstrated the potential of LbL OSCs.^[^ ^18^ ^]^ They selected PBDB‐TFS1 (**1‐3**) as the polymer donor and IT‐4F (**2‐4**) as the NFA, where THF solvent was used for processing IT‐4F on top of the PBDB‐TFS1 layer. It was proved that the PBDB‐TFS1 film almost maintained its quality when THF solvent was processed on top of it. To control the inter‐diffusion between PBDB‐TFS1 and IT‐4F, a small amount of *ortho*‐dichlorobenzene (*o*‐DCB) was introduced into THF as a cosolvent. *o*‐DCB has a much higher boiling point (b.p. 180 °C) than THF (b.p. 66 °C); thus, THF evaporates rapidly, while *o*‐DCB remains concentrated for some time during the deposition of the IT‐4F layer. This induces the penetration of IT‐4F molecules into the PBDB‐TFS1 layer; therefore, the vertical phase distribution and D/A interface can be regulated by varying the amount of *o*‐DCB. **Figure** **4**a–c depicts the schematic diagram of the morphology evolution in BHJ, LbL with only THF, and LbL films with *o*‐DCB(5%):THF(95%). The LbL morphology with the mixed solvents (Figure 4c) exhibits sufficient D/A interface along with desired vertical phase distribution; this is likely in between BHJ (Figure 4a) and LbL with 100% THF (Figure 4b). Therefore, PCEs of LbL OSCs gradually increased with increasing amounts of *o*‐DCB, from 8.11% (0% *o*‐DCB) to 11.8% (3% *o*‐DCB), and to 13.0% (5% *o*‐DCB). The increase in PCEs was mainly attributed to the improvement in short circuit current density (*J*_SC_) and fill factor (FF), as shown in Figure 4d. This result is considered important to demonstrate a noticeably higher PCE of LbL devices than that of optimized BHJ OSCs (PCE: 11.8%), triggering the recent advances of NFAs‐based LbL OSCs. After two pioneering works were reported, much effort has been made on optimizing processing solvents by several research groups.^[^ ^30^, ^32^, ^33^, ^36^, ^37^, ^38^, ^39^, ^47^, ^57^, ^60^, ^62^ ^]^

Figure 3Molecular structures of acceptor materials discussed in this review.

**Figure:**
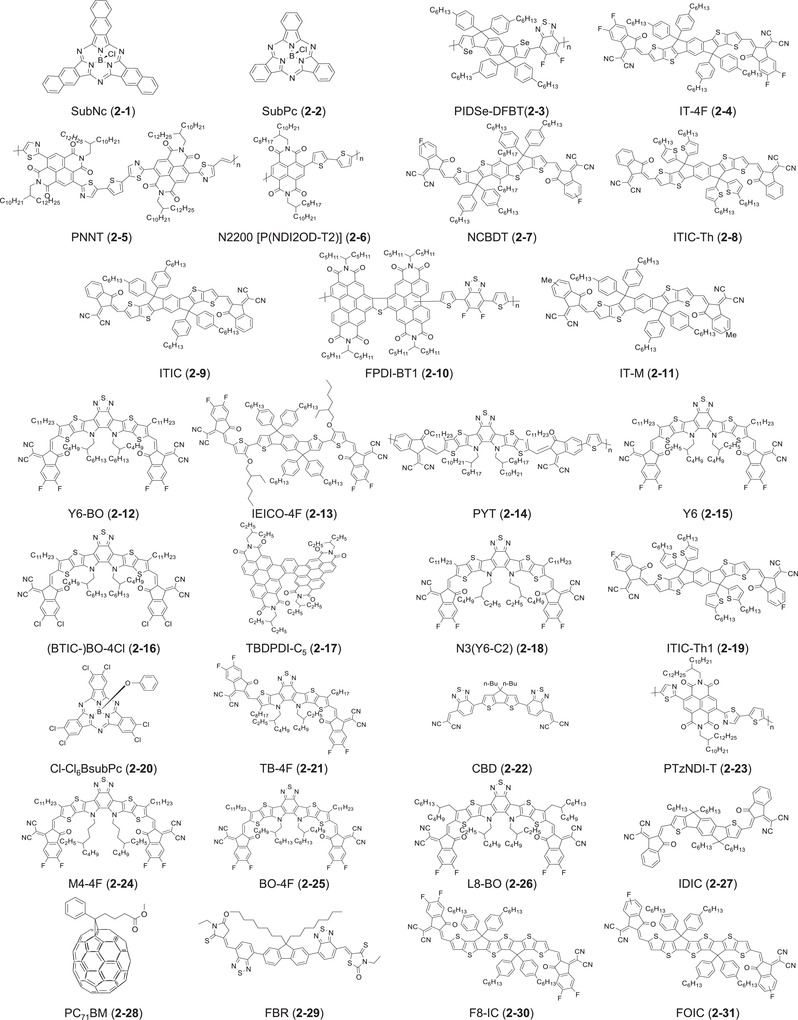

**Figure:**
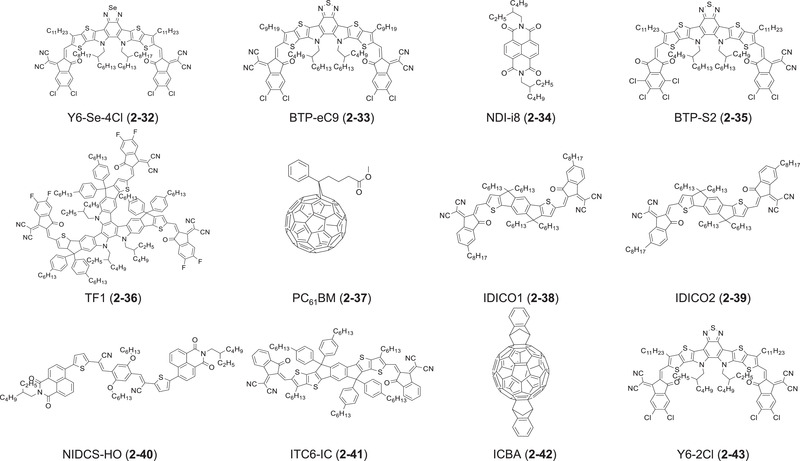

Yan et al. demonstrated the importance of the chemical structures of the underlying polymer layer by using various processing solvents including CF, THF, toluene, chlorobenzene (CB), and *ortho*‐xylene (*o*‐XY).^[^ ^34^ ^]^ The major limitation in the fabrication of LbL OSCs is the restriction in solvent selection for processing an NFA top layer; the underlying polymer film can be destroyed if NFAs are processed with nonorthogonal or high‐boiling‐point solvents. To overcome this problem, they suggested using polymer donors with temperature‐dependent aggregation (TDA) properties, which allow the polymers to disaggregate at high temperatures (≈100 °C) but aggregate at room temperature in solution. With TDA polymer donors such as PffBT4T‐2OD (**1‐21**), P3TEA (**1‐23**), and PTFB‐O (**1‐7**), the polymer films almost preserve their film quality regardless of the type of solvent, even with high‐boiling‐point solvents such as CB and *o*‐XY, because of the low solubility of TDA materials at room temperature. Figure 4e shows photographs of pristine films of TDA (P3TEA) and non‐TDA (PBDB‐T‐2Cl, **1‐14**) polymers after spin‐coating various solvents on top of them, clearly depicting that films of non‐TDA polymer are washed away with *o*‐XY and CB. Based on these results, conventional LbL OSCs using PTFB‐O/ITIC‐Th (**2‐8**) and P3TEA/IT‐4F pairs were fabricated using three different solvents (THF, CF, and toluene) to deposit the NFA layers. Interestingly, in the case of PTFB‐O/ITIC‐Th, all the LbL devices exhibited similarly high PCEs of over 11%. Notably, the PCEs of LbL OSCs were higher than those of BHJ OSCs (PCE: 10.4%). They found that the solvent of the NFAs enables inter‐diffusion between the polymer donors and NFAs; therefore, PTFB‐O and ITIC‐Th almost formed a BHJ morphology in the middle region (20–80 nm), while ITIC‐Th and PTFB‐O were mainly present in the top (80–100 nm) and bottom (0–20 nm) regions, respectively. This vertical phase distribution explains why the LbL OSCs outperform the BHJ OSCs. Wu et al. also demonstrated the importance of the solubility of polymer donors by synthesizing them with different types of side chains.^[^ ^49^ ^]^ They manipulated the solubility of naphthalenothiophene imide (NTI)‐based polymers by employing linear (PNTB6‐Cl, 1‐15) or branched alkyl chains (PNTB‐Cl, **1‐24**) at the N atom of NTI (Figure 4f). PNTB6‐Cl had stronger intermolecular interactions than PNTB‐Cl; therefore, they had different solubilities in CF: insoluble (PNTB6‐Cl) and soluble (PNTB‐Cl). When fabricating LbL OSCs by depositing an NFA N3 (**2‐18**) in CF on top of polymer donors (PNTB6‐Cl or PNTB‐Cl), only the amorphous region in the PNTB6‐Cl film was washed away while preserving the crystalline phase. However, in the case of PNTB‐Cl, both the amorphous and crystalline regions were dissolved simultaneously by the CF solvent, resulting in an unfavorable morphology (Figure 4g). As a result, PNTB6‐Cl/N3 devices exhibited much higher PCE of 17.59% as compared with PNTB‐Cl/N3 (PCE: 15.24%). These results suggest the importance of designing the chemical structures of polymer donors for producing efficient LbL OSCs with the proper choice of NFA processing solvents.

**Figure 5:**
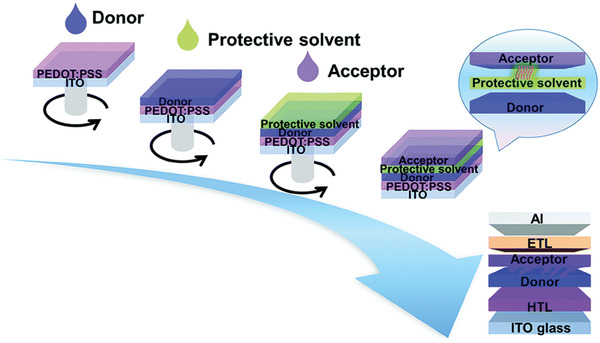
Fabrication of LbL OSCs using protective solvents. Reproduced with permission.^[^ ^54^ ^]^ Copyright 2021, Royal Society of Chemistry.

A more universal method to successfully produce a desired LbL morphology was proposed by Huang and co‐workers.^[^ ^54^ ^]^ They suggested a simple and easy method to fabricate LbL structures without any concerns regarding the damage to the underlying polymer layer by applying a layer of a protecting solvent before spin‐coating NFAs on top of the polymer donor layer (**Figure** 5). Ecofriendly solvents with nonaromatic and nonhalogenated structures, including dimethyl sulfoxide (DMSO), *N,N*‐dimethylformamide (DMF), alkane solvents (*n*‐hexane, *n*‐heptane, and *n*‐octane), methanol, ethanol, and diethyl ether, were used as protecting solvents. They fabricated conventional LbL OSCs with high‐performance D18 (**1‐12**)/N3 using CF to process NFAs. Not surprisingly, the LbL OSCs had inferior photovoltaic performance (PCE: 15.32%) compared with that of BHJ OSCs (PCE: 16.58%) because underlying D18 layers could be damaged when directly processing N3 solution in CF. When protective solvents were spin‐coated before spin‐coating the N3 CF solution, i.e., forming D18/protective solvents/N3 structures, a small amount of remaining protective solvents hindered the CF solution from dissolving the D18 layer. As a result, the PCEs of the D18/protective solvents/N3 OSCs improved compared with those without protective solvents. Among various protective solvents, *n*‐octane exhibited the best result, delivering a PCE of up to 17.52%. Based on these promising results, the universality of using protective solvents was proved by using nine different photoactive layers, including fullerene and NFAs, all of which exhibited photovoltaic performance comparable to their BHJ counterparts. Furthermore, they also suggested a solvent protection mechanism by introducing a protective factor (*δ*); please see ref. [54] for details.

LbL devices based on all‐polymer solar cells (all‐PSCs), which use p‐type and n‐type polymers as photoactive materials, have also been reported. All‐PSCs have the obvious advantages of better mechanical and thermal stability compared with small‐molecule NFA‐based OSCs.^[^ ^37^, ^38^, ^55^, ^59^ ^]^ A good example for the all‐PSCs‐based LbL approach was reported by Min et al. They fabricated conventional LbL OSCs using PBDB‐T and PYT (**2‐14**) as a polymer donor and acceptor, respectively.^[^ ^55^ ^]^ The PBDB‐T/PYT LbL structures were sequentially fabricated using PBDB‐T in CF and PYT + 1‐chloronaphthalene (CN) additive (2.5%) in CF. They found that LbL all‐PSCs also exhibited desired p–i–n vertical phase distribution. As a result, the PBDB‐T/PYT device had a higher PCE of 15.17% than BHJ device (14.06%). Cao et al. also reported LbL all‐PSCs using the same polymer donor and acceptor.^[^ ^63^ ^]^ In their report, the entire active layer of PBDB‐T/PYT+CN was thermally annealed, which gave rise to the lamellar ordering of PBDB‐T and the formation of PYT fibrils. Therefore, the device performance was further improved to a PCE of 16.05% compared with that without thermal annealing (15.17%), which is the highest PCE value for LbL all‐PSCs reported so far. A similar work on LbL all‐PSCs was also reported by Zhong group.^[^ ^36^ ^]^

Furthermore, it is easy to fabricate an inverted LbL device based on all‐PSCs because of the limited solubility of the polymer acceptors. Ma et al. successfully demonstrated inverted‐type LbL OSCs using N2200 (**2‐6**) and PBDB‐T for preparing the polymer acceptor and donor layers, respectively (ITO/zinc oxide (ZnO)/N2200/PBDB‐T/molybdenum trioxide (MoO_3_)/Ag).^[^ ^37^ ^]^ The N2200 polymer has limited solubility in CF (processing solvent for PBDB‐T) without additional heating; therefore, N2200/PBDB‐T LbL structures were successfully obtained. They found that the neat N2200 film had a highly textured surface with long‐range order of the polymer fibers, which can serve as a nanogrooved substrate. Therefore, when depositing PBDB‐T on top of the N2200 layer, enhanced molecular ordering in the PBDB‐T layer was achieved compared with BHJ structures. As a result, inverted all‐PSCs with an LbL structure delivered a PCE of 9.52%, which is significantly higher than that of BHJ devices (6.58%). Similarly, many other research groups have also reported LbL all‐PSCs using inverted‐type devices.^[^ ^30^, ^31^, ^32^, ^59^ ^]^

### 2.3 Vertical Morphology Control by Additional Treatments

The main advantage of LbL OSCs is that independent deposition of donor and acceptor layers can ensure their crystalline packing structures compared to BHJ structures. A one‐step solution process with a mixture of donor and acceptor in BHJ OSCs may interrupt the self‐assembly of each phase; therefore, it is difficult to obtain an ideal morphology with enhanced crystallinity of the donor and acceptor phases. To control the nano‐ and/or microscopic morphology of BHJ, additional treatments, such as the use of processing additives and thermal annealing, have been widely employed,^[^ ^78^, ^79^, ^80^ ^]^ but complicated dynamics and kinetics during the morphological evolution of the two phases always pose a significant challenge. However, LbL OSCs with an independent deposition process simplify the optimization of the p–i–n vertical morphology by additional treatments.

**Figure 6:**
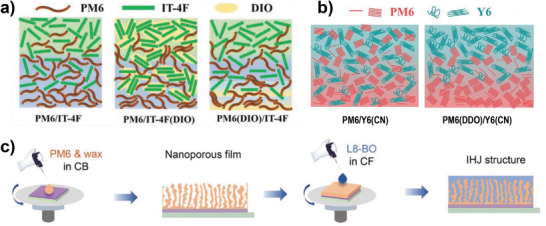
a) Schematic diagrams of film morphology in the LbL OSCs with or without DIO treatment. Reproduced with permission.^[^ ^45^ ^]^ Copyright 2021, Wiley‐VCH. b) Schematic illustration of vertical composition distribution in the LbL OSCs without and with DDO in PM6. Reproduced with permission.^[^ ^52^ ^]^ Copyright 2021, Wiley‐VCH. c) IHJ structure induced by introduction of wax additive. Reproduced with permission.^[^ ^21^ ^]^ Copyright 2021, Wiley‐VCH.

One of the most effective ways to optimize the blend morphology is the introduction of high‐boiling solvent additives such as 1,8‐diiodooctane (DIO) and CN during solution‐processing of donors and/or acceptors.^[^ ^21^, ^40^, ^44^, ^45^, ^52^, ^66^ ^]^ Chen and co‐workers systematically investigated the effect of a solvent additive DIO on the performance of LbL OSCs based on a PM6 (**1‐9**)/IT‐4F photoactive layer.^[^ ^45^ ^]^ They fabricated three LbL OSCs with or without DIO: PM6/IT‐4F, PM6/IT‐4F(DIO), and PM6(DIO)/IT‐4F. It was observed that IT‐4F exhibited ordered molecular packing in PM6/IT‐4F(DIO) because the existence of DIO (b.p. 333 °C) in the processing solvent extended the drying process of the thin film, enabling IT‐4F to have enough time to form larger crystallites. By contrast, adding DIO to a PM6 solution (PM6(DIO)/IT‐4F) had little influence on the molecular ordering of the PM6 chains because polymeric PM6 has poorer solubility in DIO compared with that of small molecular IT‐4F. They also observed that PM6/IT‐4F(DIO) exhibited the desired vertical phase separation, and IT‐4F and PM6 were enriched at the top and bottom electrodes, respectively. As a result, the LbL OSCs with PM6/IT‐4F(DIO) exhibited the best PCE of 13.70%, which was higher than those of PM6/IT‐4F (12.13%) and PM6(DIO)/IT‐4F (12.11%). **Figure** **6**a depicts a schematic diagram of the film morphology in the BHJ and LbL structures with or without DIO treatment. PM6/IT‐4F(DIO) has an optimized morphology with enhanced crystallinity and a desired vertical phase distribution.

A similar approach was also reported by Huang and co‐workers.^[^ ^66^ ^]^ LbL OSCs were formed with a PM6/Y6 (**2‐15**) photoactive layer with the addition of DIO by varying its amount (0–2 vol%) in solution; CF solvent was used for spin‐coating both PM6 and Y6 layers. In accordance with the work reported by Chen et al., they also observed improved crystallinity of the photoactive layers with the addition of DIO. It is noteworthy that the effect of different amounts of DIO was critical to the resulting vertical composition distribution. As expected, when spin‐coating Y6 in CF onto the underlying PM6 layer, Y6 molecules diffuse into the PM6 layer, forming a PM6:Y6 intermixed region because the CF solvent could swell the PM6 polymer matrix. With the addition of DIO in the Y6 solution, a thicker intermixed region was formed because the high‐boiling DIO prolonged the solvent volatilization and swelled more PM6 molecules. They found that 0.5 vol% DIO produced a desired vertical distribution: a thick intermixed region in the middle with an enrichment of PM6 and Y6 at the anode and cathode sides, respectively. Resultantly, a PCE of 16.5% was achieved in PM6/Y6 (DIO: 0.5 vol%), which is higher than that of the BHJ counterpart (15.8%). Based on these promising results, they also fabricated a ternary blend LbL OSC (PM6/Y6:PC_71_BM (**2‐28**)), achieving a PCE of 17.0%.

The vertical phase distribution was also studied by introducing two different additives into the polymer donor and NFA layers. Chen and co‐workers added 1,10‐decanediol (DDO) and CN additives to PM6 donor (in CF) and Y6 NFA (in CF), respectively, to precisely control the vertical phase distribution.^[^ ^52^ ^]^ As discussed above, high‐boiling CN (b.p. 259 °C) prolonged the drying process of the thin film, which induced tight aggregation of Y6 molecules. However, this could also cause overmixing of the Y6 and PM6 molecules, producing an undesirable BHJ‐like morphology. They discovered that the DDO additive solved the overmixing problem. The DDO additive enhanced the crystalline packing in PM6 film, thus preventing the severe diffusion of Y6 molecules; Figure 6b depicts the different vertical phase separation in LbL films with or without DDO in PM6. Consequently, the LbL OSCs using the binary additive strategy (PM6+DDO/Y6+CN) exhibited a PCE of up to 16.9%, which is higher than that of BHJ OSCs (14.9%).

Peng et al. also demonstrated the importance of the proper choice of processing additives for the underlying layer of polymer donors.^[^ ^21^ ^]^ They incorporated a trace amount of a wax additive (parafilm M, 0–1.5 wt%) into a PM6 donor solution. PM6 and wax have different surface energies (PM6: 33.0 mJ m^‐2^; wax: 27.7 mJ m^‐2^), resulting in poor miscibility. As a result, wax additives induced nanoporous structures with a size of tens of nanometers during film formation; the pore size gradually increased with increasing amounts of wax additives (Figure 6c). Then, sequential solution casting of L8‐BO (**2‐26**) in CF filled the nanoporous polymer film without significant morphological changes, maintaining the desired vertical phase distribution. They defined the structures developed in this study as interdigitated heterojunctions (IHJ). Consequentially, they achieved an outstanding PCE of 18.74% using PM6 (0.5 wt% wax)/L8‐BO based LbL devices, which is higher than that of BHJ devices (18.10%). Similar studies on finding proper additives for polymer donor and/or NFA layers have also been reported by several other research groups.^[^ ^16^, ^40^, ^41^, ^44^, ^49^, ^60^, ^63^ ^]^

Chen et al. also observed overmixing behavior between a polymer donor and NFA while depositing NFA using CF solvent, resulting in the BHJ‐like morphology.^[^ ^20^ ^]^ To produce vertically separated morphology, they introduced one more NFA, namely BTP‐S2 (**2‐35**), which has an asymmetric structure, into a PM6/BO‐4Cl (**2‐16**) system. BTP‐S2 exhibited lower miscibility with PM6 when compared with BO‐4Cl, which was quantified by the Flory‐Huggins interaction parameter *χ*.^[^ ^81^, ^82^ ^]^ Therefore, when spin‐coating the BO‐4Cl with 25 wt% BTP‐S2 onto the underlying PM6 layer, the *χ* value increased from 0.26 (only BO‐4Cl) to 0.44 (BO‐4Cl with BTP‐S2), which helps to avoid the overmixing of the D:A phase during a spin‐coating process. As a result, the PM6/(BO‐4Cl:25 wt% BTP‐S2) ternary LbL devices showed a PCE of 18.16%, which was higher than that of the binary one with PM6/BO‐4Cl containing no BTP‐S2. Similar approaches for LbL OSCs using ternary blends to obtain optimal blend morphology and absorption properties have been reported.^[^ ^65^, ^68^, ^69^ ^]^

Thermal annealing is also an effective strategy to regulate the crystalline structures and vertical phase distribution of LbL OSCs. Yang et al. developed inverted‐type LbL OSCs with a configuration of ITO/ZnO:poly(ethyleneimine) (PEI)/PM6/Y6/MoO_3_/Ag. Here, an active layer of PM6 (CB solution) and Y6 (CF solution) was sequentially deposited using a slot‐die coating method.^[^ ^57^ ^]^ In this method, it is important to control the solvent evaporation kinetics by thermal annealing treatment. First, the underlying PM6 layer was thermally annealed at different temperatures (70 °C, 100 °C, and 130 °C), followed by the deposition of a Y6 layer. The optimized annealing temperature was 70 ℃ (PCE: 9.69%) because higher annealing temperatures of 100 ℃ (PCE: 9.41%) and 130 ℃ (PCE: 9.22%) caused excessive crystallization of PM6, which prevented the formation of an intermixed phase. They also examined the thermal annealing effect after the deposition of both layers; the PM6/Y6 LbL photoactive layer was thermally treated varying the annealing temperature, and the best PCE of 14.42% was obtained at a thermal annealing temperature of 135 ℃. The optimized conditions balanced the solvent evaporation of the PM6 and Y6 films, resulting in the desired interpenetration network of PM6 and Y6.

**Figure 7:**
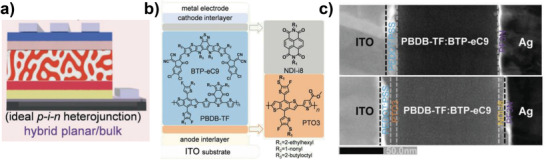
a) Schematic illustration of hybrid planar/bulk devices with ideal p–i–n heterojunction formation, b) the corresponding chemical and device structures, and c) cross‐sectional transmission electron microscope images of the devices. Reproduced with permission.^[^ ^19^ ^]^ Copyright 2021, Wiley‐VCH.

Hou et al. demonstrated that a hybrid planar/bulk heterojunction structure can be another simple way to control ideal p–i–n structures.^[^ ^19^ ^]^ They suggested a p‐type polymer/D:A BHJ/n‐type small‐molecule structure for constructing ideal p–i–n LbL devices (**Figure** **7**a). For the first layer of the p‐type polymer, they designed a benzodithiophene‐*alt*‐thiophene polymer named PTO3 (**1‐22**) with a short alkyl ester substituent on thiophene. The polymer showed a strong solution pre‐aggregation effect; it could only be dissolved in hot *o*‐DCB (at 120 °C). This made it possible to cast a BHJ layer of PM6:BTP‐eC9 (**2‐33**) in CF on top of PTO3 without damaging the underlying PTO3 layer. On the cathode side, a small molecular acceptor, NDI‐i8 (**2‐34**), was deposited by thermal evaporation to improve electron transport and block hole transfer to the cathode, which produced a final hybrid heterojunction with a favorable vertical component distribution (Figure 7b,c). PTO3 and NDI‐i8 layers have a different hole (*μ*_h_) and electron (*μ*_e_) mobilities: PTO3 has high *μ*_h_ and low *μ*_e,_ and NDI‐i8 has high *μ*_e_ and low *μ*_h_. This facilitated selective hole (through PTO3) and electron (through NDI‐i8) transport to the anode and cathode, respectively. As a result, the hybrid LbL device of PTO3/PM6:BTP‐eC9/NDI‐i8 exhibited a PCE of 18.5%, which was significantly higher than that of the BHJ device without the PTO3 and NDI‐i8 layers (17.4%).

## 3 Importance of Exciton Diffusion Length for Efficient LbL OSCs

During the past three decades, BHJ structures have dominated OSC research compared with LbL‐processed PHJ and/or PPHJ structures because the limited *L*_D_ (≈10 nm) of organic semiconductors inevitably requires the formation of intermixed D/A interfaces to effectively produce free charges resulting from exciton dissociation.^[^ ^83^ ^]^ Remarkable advances in LbL devices have been triggered by the development of NFAs composed of fused‐ring structures. Several studies have revealed that NFAs have longer *L*_D_s than fullerenes, enabling optimal domain sizes of 20–50 nm in BHJ blends.^[^ ^14^, ^15^, ^16^ ^]^ In addition, long‐range FRET from donor to acceptor layers in LbL structures effectively occurs when NFAs are used instead of fullerene derivatives, resulting in the formation of a charge‐transfer (CT) state at the D/A interfaces.^[^ ^16^, ^29^, ^41^ ^]^ These provide new opportunities for further improvements of LbL OSCs to yield higher PCEs than those of BHJ structures. To understand the reason for long *L*_D_ in NFAs, the exciton diffusion mechanism is briefly discussed here; for a detailed explanation, please refer to previous reports.^[^ ^83^, ^84^, ^85^ ^]^

Exciton diffusion can be regarded as random motion of neutral particles. In the literature, the average *L*_D_ of organic semiconductors is defined as,^[^ ^83^ ^]^

(1) $$L_{D}=\sqrt{ZD\tau }\;$$

where *Z* is the dimensionality of the exciton diffusion (1‐, 2‐, or 3D diffusion), *D* is the exciton diffusion coefficient with a unit of cm^2^ s^–1^, and *τ* is the exciton lifetime. Because a smaller bandgap generally exhibits a decreased *τ* according to the energy gap law, *D* can be considered as the main factor for determining *L*_D_.^[^ ^14^, ^86^ ^]^

Upon absorption of photons, singlet excitons are mostly generated within donor and acceptor phases, and the long‐range diffusion of these mainly occurs via FRET.^[^ ^83^, ^85^ ^]^ FRET is a nonradiative energy transfer via a dipole–dipole interaction between donor and acceptor; therefore, an exciton can be relocated from an excited donor to an acceptor via FRET. Here, it is noteworthy that donor and acceptor refer to neighboring donor molecules (or acceptor ones) in a single phase of p‐type (or n‐type) molecules, both of which can have the same chemical structure. The relationship between *L*_D_ and FRET efficiency is expressed as,^[^ ^83^ ^]^

(2) $$L_{D}=\sqrt{\tau_{f}D}\;=\frac{1}{d^{2}\sqrt{6}}\;\;\sqrt{R_{0}^{6}\frac{\tau_{f}}{\tau_{0}}}=\frac{1}{d^{2}\sqrt{6}}\;\sqrt{\frac{9\phi_{\mathrm{PL}}\kappa^{2}}{128\pi^{5}n^{4}}\frac{\tau_{f}}{\tau_{0}}J}$$

where *R*_0_ is the Förster radius (typically 1–10 nm), *τ*_0_ and *τ*_f_ are the intrinsic exciton lifetime and photoluminescence (PL) lifetime in a solid film, respectively, *κ* ^2^ is the dipole orientation factor, *d* is the intermolecular distance, *Φ*_PL_ is the photoluminescence quantum yield, and *J* is the spectral overlap integral between the emission of the donor and absorption of the acceptor molecules. In particular, it has been regarded that optimizing *Φ*_PL_ and *J* is especially important for increasing *L*_D_.^[^ ^83^, ^87^ ^]^ Therefore, it is necessary to develop conjugated materials with small Stokes shifts for efficient self‐FRET between donor molecules in the D phase and between acceptor molecules in the A phase, together with high *Φ*_PL_, to increase *L*_D_.

The inhomogeneity in the excitonic density of states (DOS) must also be considered, which is caused by local variations in the electronic environment.^[^ ^83^, ^84^, ^85^ ^]^ Conjugated materials in the active layers of OSCs are usually semicrystalline (or fully amorphous), with ordered molecules embedded in a disordered medium, causing a segment of conjugated materials to be in different environments with respect to their neighbors. This increases the distribution of DOS, the width of which is described by the energetic disorder *σ*. Excitons created within the DOS migrate toward sites of lower energy until they reach the occupied density of states (ODOS) that are centered around the thermal equilibrium energy, and the center of the ODOS is shifted by ‐*σ* ^2^/*kT* with respect to the center of the DOS. Further exciton diffusion requires thermal activation and/or FRET; otherwise, they are trapped and recombined. In this regard, *L*_D_ can be increased by reducing the *σ* of conjugated materials because a small *σ* makes DOS and ODOS closer, which provides more pathways for excitons to migrate.

Overall, *Φ*_PL_ and *J* need to be increased and *σ* reduced, to increase *L*_D_. Fullerene derivatives such as PC_61_BM (**2‐37**) and PC_71_BM mainly absorb photons in the UV region without noticeable PL (low *Φ*_PL_), leading to very low *J*. This indicates that exciton diffusion in fullerenes via FRET is negligible. Alternatively, NFAs have rigid structures imparted by the fused ring in the backbone, resulting in a strong overlap of absorption and PL spectra (high *J*) to increase the *L*_D_ via self‐FRET.^[^ ^14^, ^16^ ^]^ Moreover, the rigid and crystalline structures of NFAs can reduce *σ* compared to fullerenes, enhancing the density of energetically accessible transfer sites. Therefore, excitons in NFAs can be sustained through efficient self‐FRET throughout their lifetimes. Additionally, the use of NFAs instead of fullerenes in LbL p–i–n structures facilitates long‐range exciton diffusion via FRET from donor to acceptor layers because NFAs usually have a large spectral overlap of absorption with the PL of donor polymers. The transferred excitons in the NFA layer subsequently form a CT state and dissociate by hole transfer from the NFA to the donor molecules at the D/A interfaces.^[^ ^16^, ^29^, ^41^ ^]^

**Figure 8:**
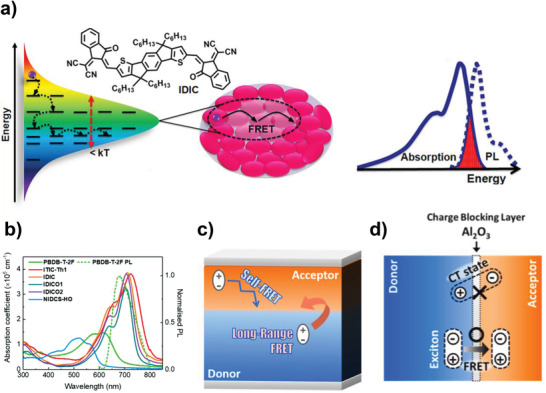
a) Enhanced exciton diffusion length of IDIC by self‐FRET and low *σ*. Reproduced with permission.^[^ ^14^ ^]^ Copyright 2019, American Chemical Society. b) Absorption spectra of NFAs and PL spectrum of polymer donor (PBDB‐T‐2F), c) exciton diffusion mechanism via FRET, and d) long‐range energy transfer from polymer donor to NFA layers in LbL OSCs. Reproduced with permission.^[^ ^16^ ^]^ Copyright 2021, Elsevier.

Several research groups claimed that the increased *L*_D_ of NFAs and long‐range FRET from donor to acceptor phases can produce high‐performance LbL OSCs with the use of NFAs.^[^ ^14^, ^15^, ^16^, ^41^, ^60^, ^72^, ^88^ ^]^ In 2019, the Hodgkiss group examined the exciton diffusion behavior of an NFA molecule, IDIC (**2‐27**), which has a fused ring structure based on an indacenodithiophene moiety.^[^ ^14^ ^]^ They utilized the exciton annihilation method to measure *D* by ultrafast transient absorption spectroscopy (TAS) as a function of excitation density; the advantage of this method is that the experiment is carried out by using IDIC bulk films without an exciton quenching interface. Assuming a Förster radius for exciton–exciton annihilation of 4.8 nm, a *D* of 2 × 10^–2^ cm^2^ s^‐1^ was obtained for IDIC, which is much higher than those of other classical organic semiconductors including fullerene acceptors, for example, PC_71_BM (*D* = 1.6 × 10^–4^ cm^2^ s^‐1^), P3HT (**1‐2**, *D* = 1.8 × 10^–3^ cm^2^ s^‐1^), poly(*p*‐phenylene vinylene) derivatives (*D* = 0.3–3 × 10^–3^ cm^2^ s^‐1^), and PCPDTBT (**1‐25**, *D* = 3 × 10^–3^ cm^2^ s^‐1^). Using Equation (1), with the assumption of 1D diffusion, the *L*_D_ of IDIC was calculated to be 16 nm. This value is exceptionally high compared with those of other organic semiconductors, and few materials exhibit an *L*_D_ > 10 nm for 1D diffusion. The main reason for the high *L*_D_ of IDIC is the strong spectral overlap of its absorption and PL spectra (with a small Stokes shift), which arises from the structural rigidity of the fused‐ring structure of IDIC, enabling long‐range exciton diffusion via self‐FRET (**Figure** **8**a). Furthermore, the crystalline structure of IDIC molecules in a solid film leads to a low *σ* of 10–23 meV, providing more pathways for excitons to migrate.

Based on the above result, Hodgkiss and co‐workers investigated the photovoltaic performance of LbL OSCs by using PBDB‐T‐2F (PM6) as a polymer donor and various NFAs, including ITIC‐Th1 (**2‐19**), IDIC, IDICO1 (**2‐38**), IDICO2 (**2‐39**), and NIDCS‐HO (**2‐40**).^[^ ^16^ ^]^ The first four NFAs have fused‐ring structures, giving rise to low Stokes shift (<75 nm) and lower bandgap compared with PBDB‐T‐2F. Alternatively, NIDCS‐HO having a benzene ring as a central core exhibited a larger Stokes shift (> 133 nm) and higher bandgap than that of PBDB‐T‐2F. They fabricated LbL OSCs using DCM as a processing solvent for NFAs to minimize the diffusion of NFAs into a PBDB‐T‐2F bottom layer, which readily enabled the examination of exciton diffusion behavior. As expected, PBDB‐T‐2F/(ITIC‐Th1, IDIC, IDICO1, and IDICO2) LbL OSCs exhibited high photovoltaic performance of 9–11% PCEs, whereas the device with NIDCS‐HO yielded poor photovoltaic performance with a PCE of 2.57%. This must be directly related to the long *L*_D_ of fused‐ring‐structured ITIC‐Th1 (*L*_D_ = 10.6–56.6 nm), IDIC (*L*_D_ = 35 nm), IDICO1 (*L*_D_ = 20.3 nm), and IDICO2 (*L*_D_ = 23.5 nm) via self‐FRET. By contrast, exciton diffusion via self‐FRET in NIDCS‐HO was limited because of its high Stokes shift, leading to a short *L*_D_ of 8.7 nm. In addition to the different *L*_D_ values of the NFAs, long‐range FRET between the polymer donor and NFA layers also contributed to the high PCEs of the LbL OSCs (Figure 8b,c). To confirm the presence of D–A FRET, they inserted a thin charge‐blocking layer of Al_2_O_3_ (≈7 nm) between the polymer donor (PBDB‐T‐2F) and NFA (IDIC) layers, which allowed D/A FRET but prevented direct exciton dissociation at the D/A interface (Figure 8d). From the TAS measurements, they observed the appearance of IDIC singlet excitons by selective excitation of PBDB‐T‐2F at 560 nm. Subsequently, the excitons in the IDIC layer dissociated to form a CT state via hole transfer at the D/A interfaces.

Similar phenomena were also reported by other groups.^[^ ^29^, ^41^ ^]^ Kim et al. compared long‐range FRET characteristics between polymer donors (PBDB‐T‐2F) and acceptor layers in an LbL architecture using a series of acceptors, including ITIC (**2‐9**), IDIC, IT‐4F, and PC_71_BM. From time‐resolved PL measurements, it was observed that efficient FRET and CT state formation at the D/A interface occurred in NFAs (ITIC, IDIC, and IT‐4F)‐based LbL devices, but not in the PC_71_BM‐based device, which was also explained by the different extents of spectral overlap between the PL of the donor and absorption of acceptors (no spectral overlap in a PC_71_BM and PBDB‐T‐2F pair). As a result, all the LbL OSCs based on the NFAs exhibited high PCEs of up to 10.1%, but the device with PC_71_BM had a PCE of only 3.41%.

## 4 Large Area LbL OSCs and Device Stability

Although remarkably high PCEs of over 18% have been successfully achieved at the lab‐scale using small area BHJ and LbL devices, up‐scaling the OSCs to enter large‐scale production for industrial applications still remains a challenge. The interpenetrating D/A network structure required in BHJ is generally created by spontaneous nanoscale phase separation. The optimal BHJ morphology is metastable and further moves toward a thermodynamic equilibrium state to form a gradual phase separation, thereby degrading device performance and operational stability. The difficulty in regulating the BHJ morphology makes it inadequate for obtaining large‐area OSCs. Sequential blade coating (SBC) may offer an affordable solution for a scalable printing technique to produce ideal p–i–n PPHJ photoactive films, which is also expected to bridge the efficiency gap between lab‐scale cells and large‐scale devices (and modules). Compared with the spin‐coating process, the SBC printing process is a simple, environmentally friendly, and low‐cost method for large‐area film fabrication with high reproducibility and a high material utilization rate. It can be easily applied to high‐throughput R2R industrial manufacturing of OSCs. High‐quality photoactive layers with controllable thicknesses can be obtained by adjusting the blade speed and substrate temperature. In addition, for the practical application of OSCs, long‐term device stability related to morphology evolution is also a critical factor to be considered. Furthermore, mechanical properties are also crucial for the realization of large and flexible OSCs.

**Figure 9:**
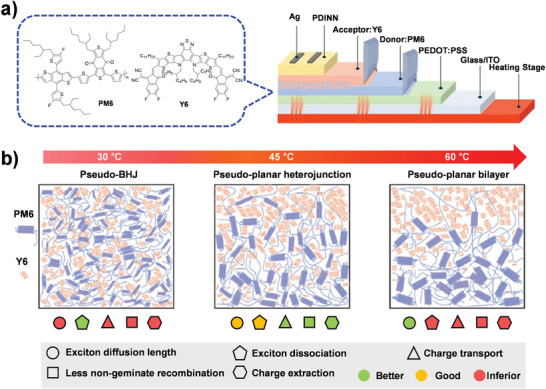
a) Schematic diagram of blade coating approach with different baseplate temperatures and b) evolution of morphological properties as a function of baseplate temperature. Reproduced with permission.^[^ ^61^ ^]^ Copyright 2021, Wiley‐VCH.

To give real insight into which approach is more suitable for industrialization, Min et al. studied and compared the BHJ and LbL processing strategies based on the J71 (**1‐26**):ITC6‐IC (**2‐41**) system using a doctor‐blade coating technology, investigating the relationships between blend morphology, photophysical dynamics, photovoltaic parameters, and device stability.^[^ ^89^ ^]^ The LbL active layer with suitable vertical phase separation and highly crystalline domains preserved the interfacial exciton harvesting and suppressed the charge recombination with reduced traps and defects. The pronounced diode property with low leakage current in the dark *J–V* characteristics is a strong advantage of LbL p–i–n devices.^[^ ^37^ ^]^ They also found that the SBC approach could reduce the energy loss in the J71:ITC6‐IC system, obtaining an open‐circuit voltage (*V*_OC_) of 950 mV with an energy loss of 0.72 eV for the BHJ device and a *V*_OC_ of 968 mV with a smaller energy loss of 0.702 eV for the LbL device. The vertical phase gradation in the LbL blend can effectively narrow the shape of the DOS with the up‐shifted electron quasi‐Fermi level of the acceptor and the down‐shifted hole quasi‐Fermi level of the donor, inducing better Fermi level alignment with the electrodes.^[^ ^90^ ^]^ The LbL OSCs exhibited a significantly smaller nonradiative energy loss of 0.355 V compared with that of the BHJ devices (0.371 V), suggesting that the LbL approach plays a significant role in reducing nonradiative recombination and thus increasing *V*_OC_. The authors also investigated the stability of the BHJ and LbL blends, including their photostability, thermal stability, and mechanical stability. In a nitrogen glovebox at room temperature, the BHJ device showed a light‐induced PCE degradation down to 68% within 500 h, whereas the LbL device showed a lower PCE loss down to ≈85% during the same period. The larger PCE drop in BHJ OSCs is interpreted to originate from their morphological instability to form large domains, resulting in an obvious burn‐in loss. In addition to light, accumulated heat during device operation is unavoidable and changes the optimized blend morphology, further leading to PCE loss. Better thermal stability was also measured for the LbL devices compared with the BHJ blends; the LbL devices maintained 88% of their initial performance after baking at 120 ℃ under an inert atmosphere for 1500 h (81% for BHJ devices), probably because of the highly stable film morphology. They also measured the PCEs of BHJ and LbL devices based on the configuration of polyethylene terephthalate (PET)/ITO‐metal‐ITO (IMI)/PEDOT:PSS/active layer/PDINO/Al as a function of bending cycles with a radius of 6 mm. The LbL‐bladed device maintained 92% of its initial PCE, whereas the BHJ devices showed approximately 85% of its initial PCE after 2000 bending cycles. The LbL processing approach primarily acts as a plasticizer to enable the ripening of the domains with aggregation of molecules to induce vertical p–i–n phase separation, thus making it possible to achieve a more rational control of vertical stratification to obtain high mechanical stability, demonstrating a new potential of flexible OSCs. Min et al. also investigated the temperature‐controlled vertical blend morphology in the LbL‐processed PM6:Y6 OSCs.^[^ ^61^ ^]^ During a doctor‐blade coating process, different baseplate temperatures from 20 °C to 60 °C were employed (**Figure** **9**a). They found out that different baseplate temperatures produced different morphology evolution: BHJ‐like, p–i–n‐like, and bilayer‐like morphologies were systematically controlled by changing the baseplate temperatures of low (20 °C and 30 ℃), medium (40 °C and 45 °C), and high temperatures (50 °C and 60 °C), respectively (Figure 9b). The rate of solvent evaporation influences the diffusion of Y6 molecules into the underlying PM6 layer. The devices processed at 45 °C exhibited optimal exciton dissociation and charge transport/collection efficiencies. As a result, the LBL OSCs processed at a baseplate temperature of 45 °C delivered the highest PCE of 16.17%, which is significantly higher than those processed at 30 °C (12.78%) and 60 °C (13.59%). This suggests the importance of precisely controlling the baseplate temperature for vertical morphology control in a doctor‐blade coating, which may provide a nice tip toward the rational optimization of device performance in the lab‐to‐fab transition

Chen reported a highly efficient large‐area (1.05 cm^2^) PPHJ OSC of a ternary blend of PM6:IT‐4F:ICBA (**2‐42**) by SBC with a PCE of 14.25%, which is even higher than that of the BHJ devices (13.73%).^[^ ^91^ ^]^ Furthermore, the LbL devices exhibited better thermal stability as compared with the BHJ devices. After thermal annealing at 150 ℃ for 40 h, the LbL OSCs had 87% of the original PCE, whereas that of the BHJ devices decreased to 81% of the initial value. The optimal morphology of BHJ films is generally metastable, where the self‐aggregation of the donor and acceptor with increased phase separation leads to unbalanced carrier transport with degradation in device performance. The p–i–n PPHJ structure is formed by depositing the donor and acceptor layers separately, and its favorable phase separation with purer phases is expected to be important for the thermodynamically stable morphology, which is promising toward realizing superior stability of OSCs. Similar results of higher thermal stability of LbL OSCs by spin casting (compared with spin‐coated BHJ devices) have also been reported.^[^ ^37^, ^45^, ^66^, ^69^, ^74^ ^]^ These results emphasize that favorable phase separation with purer phases is important for achieving thermodynamically stable blend morphology and realizing highly efficient OSCs with superior stability.

**Figure 10:**
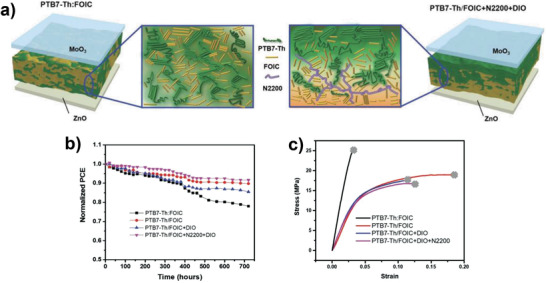
a) Schematic illustration of morphological evolution in PTB7‐Th:FOIC and PTB7‐Th/FOIC+N2200+DIO films, b) normalized PCEs as a function of storage time in nitrogen, and c) stress‐strain curves of PTB7‐Th:FOIC, PTB7‐Th/FOIC, PTB7‐Th/FOIC+DIO, and PTB7‐Th/FOIC+N2200+DIO devices. Reproduced with permission.^[^ ^43^ ^]^ Copyright 2020, Wiley‐VCH.

Ma fabricated highly efficient inverted n–i–p LbL OSCs of ITO/ZnO/FOIC (**2‐31**) (x% N2200)/PTB7‐Th (**1‐10**)/MoO_3_/Ag by SBC technique in ambient environment (**Figure** **10**a).^[^ ^43^ ^]^ The un‐encapsulated SBC‐processed OSCs maintained ≈90% of the original PCE after storage for 30 days in a nitrogen atmosphere, showing higher stability compared with the BHJ devices (≈78%) (Figure 10b). Moreover, the mechanical stability of the LbL devices was significantly enhanced compared with that of the BHJ devices. The BHJ film exhibited no obvious plastic deformation region and broke at a short elongation, whereas the LbL films showed cracks at a much longer elongation. The crack‐onset strain (COS) was measured to be 3.1% and 18.5% for the BHJ (PTB7‐Th:FOIC) and LbL (PTB7‐Th/FOIC) films, respectively, showing a six‐fold higher COS for SBC‐processed LbL structures (Figure 10c). They claimed that the SBC‐processing method provides a suitable D/A interface area and purer phases, resulting in a thermodynamically stable morphology with improved long‐term stability and excellent mechanical resilience with high elongation.

**Figure 11:**
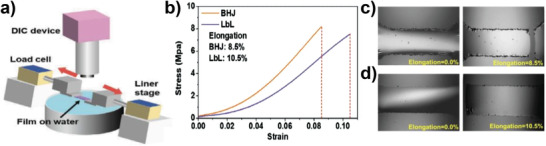
a) Schematic illustration of tensile test setup based on floated ultrathin films, b) stress‐strain curves of BHJ and LbL films, optical microscope images of c) BHJ and d) LbL processed films when the films were under different strains. Reproduced with permission.^[^ ^55^ ^]^ Copyright 2021, Wiley‐VCH.

**Figure 12:**
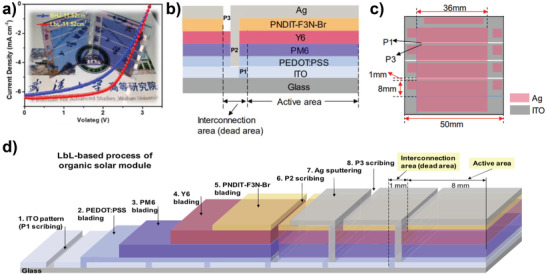
a) Solar modules based on LbL PM6/Y6 films with an active area of 11.52 cm^2^ and *J–V* curves of the best‐performing BHJ and LbL‐based devices, b) schematic illustration of section for the solar modules with the large area, c) the actual configuration of solar modules based on four series‐connected single cells of 8 mm × 36 mm (active area : 11.52 cm^2^), and d) LbL process flow diagram for fabricating large‐area solar modules. Reproduced with permission.^[^ ^22^ ^]^ Copyright 2020, Elsevier.

Min et al. also studied the mechanical stability of PBDB‐T/PYT‐based all‐PSCs via the LbL doctor‐blading technique.^[^ ^55^ ^]^ Excellent mechanical stability is a great merit of all‐PSCs owing to the formation of large plastic areas and good interchain networks. To compare the mechanical flexibility of the BHJ and LbL structures, the strain–stress curves of the blade‐coated BHJ and LbL films were recorded by tensile testing with a photoactive film floating on water (**Figure** **11**a). The LbL PBDB‐T/PYT film showed a superior ductile nature, exhibiting a break at a high elongation of 10.5% (BHJ films broke at an 8.5% elongation) (Figure 11b–d). The better mechanical properties of the LbL films probably resulted from the high molecular ordering with an optimized vertical phase morphology. They also reported an LbL SBC approach for the fabrication of large‐area OSCs and modules.^[^ ^22^ ^]^ The solar cells and modules were fabricated with a conventional device architecture of ITO/PEDOT:PSS/active layer/poly[[2,7‐bis(2‐ethylhexyl)‐1,2,3,6,7,8‐hexahydro‐1,3,6,8‐tetraoxobenzo[lmn][3,8]phenanthroline‐4,9‐diyl]‐2,5‐thiophenediyl[9,9‐bis[3′((*N,N*‐dimethyl)‐N‐ethylammonium)]‐propyl]‐9H‐fluorene‐2,7‐diyl]‐2,5‐thiophenediyl] (PNDIT‐F3N‐Br)/Ag, and the active layer and interface layers were prepared by blade coating in air. They measured a high PCE of 16.35% in the small‐scale (active area of 0.04 cm^2^) LbL PM6:Y6 OSCs. In addition, a noticeably high PCE of 11.86% was achieved for a large‐area (11.52 cm^2^) solar module with a geometrical fill factor of over 90%, which is the highest efficiency of large‐area solar modules reported so far (**Figure** **12** and **Table** **2**). The LbL‐blade‐coated devices showed higher PCEs than BHJ devices for a wide range of highly efficient photovoltaic systems including PM6:Y6‐2Cl (**2‐43**), PTQ10 (**1‐27**):Y6, and PM6:Y6‐C2 (**2‐18**), suggesting the universality of this strategy. Finally, the authors emphasized the unique advantages of controllable p–i–n morphology, enhanced light absorption in the film, good charge transport and extraction properties, and significant universality of LbL‐processed OSCs compared with their conventional BHJ counterparts.

**Table 2:** Photovoltaic properties of large‐area NFAs‐based LbL OSCs by sequential blade coating

Active layer	Processing condition	Stability	*V*_OC_ [V]	*J*_SC_ [mA cm^‐2^]	FF [%]	PCE [%]	Refs.
PM6(**1‐9**)/IT‐4F(**2‐4**)	ITO/PEDOT:PSS/D (*o*‐XY)/ A (*o*‐XY)/PNDIT‐F3N/Ag Device area : 1.00 cm^2^		0.84	20.00	68.00	11.40	^[^ ^96^ ^]^
PTQ10(**1‐27**)/IDIC(**2‐27**)	ITO/PEDOT:PSS/D (CF)/A (CF)/PDINO/Al Device area : 1.00 cm^2^	90% at 100 ℃ for 10 h	0.97	15.89	67.90	10.42	^[^ ^97^ ^]^
J71(**1‐26**)/ITC6‐IC(**2‐41**)	ITO/PEDOT:PSS/D (CF)/A (CF)/PDINO/Al Device area : 1.00 cm^2^	90% at 120 ℃ over 1500 h /92% after 2000 bending cycle	0.97	16.85	70.10	11.47	^[^ ^89^ ^]^
PM6(**1‐9**)/IT‐4F(**2‐4**):ICBA(**2‐42**) (0.9:0.1)	ITO/ZnO/D (CB)/A (CB with 0.5vol% DIO)/PDNIO/Al Device area : 1.05 cm^2^	87% at 150 ℃ for 40 h	0.88	21.25	76.55	14.25	^[^ ^91^ ^]^
PM6(**1‐9**)/Y6(**2‐15**)	ITO/PEDOT:PSS/D (CF)/A (CF)/PNDIT‐F3N‐Br/Ag Module area : 3.30 cm^2^		2.49	8.72	63.92	13.88	^[^ ^22^ ^]^
PM6(**1‐9**)/Y6(**2‐15**)	ITO/PEDOT:PSS/D (CF)/A (CF)/PNDIT‐F3N‐Br/Ag Module area : 11.52 cm^2^		3.20	6.41	57.85	11.86	^[^ ^22^ ^]^

Slot‐die coating is also considered to be a scalable printing technique suitable for large‐scale and high‐throughput R2R industrial mass production. Several research groups have fabricated highly efficient LbL OSC devices using slot‐die coating.^[^ ^92^, ^93^, ^94^, ^95^ ^]^ To induce a vertical phase separation, the LbL slot‐die coating was investigated by Yang et al.^[^ ^57^ ^]^ By using two kinds of different boiling point solvents (CB/CF), the donor (PM6) layer was cast using a high‐boiling CB, and the acceptor (Y6) solution in CF was slot‐die coated directly on top of the PM6 layer. The high‐boiling solvent remains in the donor layer during the deposition of the acceptor layer, so the donor and acceptor blend well (donors move upward and acceptors move down) to achieve an ideal vertical phase distribution. Finally, the LbL slot‐die coated OSCs using two solvents exhibited a PCE up to 14.42%, with a *V*_OC_ of 0.80 V, a *J*_SC_ of 25.28 mA cm^–2^, and a FF of 71.3%, successfully demonstrating the significant potential of LbL slot‐die coating fabrication.

## 5 Conclusions and Prospects

After the appearance of efficient NFAs, remarkable advances have been made toward the development of sequential LbL OSCs, successfully demonstrating a similar photovoltaic performance (over ≈18%) as BHJ OSCs. Benefitting from p–i–n morphological control by sequential D/A layer deposition, a significant potential for large‐area R2R production of LbL OSCs by sequential blade coating has also been demonstrated, showing over 11% PCE in the large‐area (≈11 cm^2^) solar modules. The enhanced *L*_D_ of NFAs via self‐FRET compared with fullerene derivatives provided new opportunities for the optimization of LbL PPHJ devices owing to efficient exciton separation in the PHJ and/or PPHJ, similar to BHJ structures. Different types of LbL devices have been successfully demonstrated, including polymer donor and small molecular acceptor pairs, all‐PSCs, and ternary OSCs. Vertical phase separation in LbL devices has been optimized by proper solvent selection (orthogonal/semiorthogonal solvents), processing additives, post treatments (thermal annealing, etc.), and protecting solvent treatment. The p–i–n vertical phase separation with enrichment of the donor near the anode and enrichment of the acceptor near the cathode with enhanced D and A layer crystalline ordering improves charge transport and extraction with reduced electron–hole recombination. Benefitting from these morphological advantages, smaller energy loss and higher thermal and mechanical device stability have also been obtained, suggesting another promising feature of LbL PPHJ devices. Although great potential and significant progress have been reported in sequential LbL OSCs, several challenges remain for their further optimization and successful industrialization.

First, vertical phase separation is a significant advantage in LbL devices; however, it is still difficult to fine‐control the ideal p–i–n morphology. Hou et al. tailored a hybrid planer/bulk heterojunction structure depositing a PBDB‐TF:BTP‐eC9 blend between p‐type PTO3 and n‐type NDI‐i8 layers to achieve the best PCE of 18.5%, demonstrating that the hybrid structure is a promising strategy to easily modulate the vertical D/A distribution.^[^ ^19^ ^]^ In addition, cross‐linking the underlying layer can be an effective strategy for easily producing desired vertical phase distribution because a cross‐linked layer would have solvent resistance against various solvents. The degree of diffusion of NFAs can also be regulated by controlling the crosslinking density. LbL OSCs with cross‐linking have been reported in fullerene‐based OSCs but have not been used in NFA‐based LbL OSCs.^[^ ^98^, ^99^, ^100^, ^101^, ^102^ ^]^ To realize the practical applications of LbL OSCs, reproducible p–i–n morphological control at a large‐area module needs to be studied further. Second, although the conventional device architecture of anode/D/A/cathode has been widely studied in LbL OSCs, inverted devices have been rarely studied because of solubility issues. Because most NFAs with good solubility can be easily washed away during the casting of donor polymers on the top of NFAs, it is difficult to find an appropriate semi‐orthogonal solvent for the LbL processing of inverted OSCs. It is necessary to investigate inverted devices and more efficient device structures, such as LbL tandem devices, which may suggest new opportunities to further optimize the performance and operational stability of LbL OSCs. Third, most previous studies on LbL devices have mainly focused on the photovoltaic properties under 1 sun condition. As discussed above, LbL PPHJ structures have several advantages compared with BHJ structures including a low dark current. Under indoor dim light conditions, the minimization of leakage (or dark) current with suppressed trap‐assisted recombination is important to improve the diode characteristics with a high FF because the generated carrier density is small.^[^ ^103^ ^]^ For this reason, suppressing the dark current using the p–i–n vertical phase separation in LbL OSCs is beneficial for further optimization of indoor OSC devices. Fourth, the development of ecofriendly LbL processing using green solvents suitable for industrial‐scale R2R production is an imperative research focus for the lab‐to‐fab transition. Currently, toxic and hazardous halogenated solvents (CB and CF) and processing additives (CN and DIO) are commonly used to fabricate LbL devices. However, these toxic solvents pose serious risks to human health, safety, and the environment. The management of these hazardous solvents and waste recovery are major concerns in industry, and material/processing optimization needs to be considered carefully for environmentally benign R2R LbL processing. Finally, more efforts should be directed toward improving the long‐term stability of the devices to ensure the practical viability of LbL OSCs. Although the higher thermal stability of LbL PPHJ devices compared with BHJ devices has been claimed, more systematic investigations are required on the operational device stability, including photo, thermal, and environmental stabilities under light soaking at high temperatures and humid conditions (i.e., 85 ℃ and 85% humidity). These considerations are expected to advance sequential LbL OSC technology a step forward toward its eventual commercialization.

## Conflict of Interest

The authors declare no conflict of interest.
